# Socioeconomic status and environmental noise exposure in Montreal, Canada

**DOI:** 10.1186/s12889-015-1571-2

**Published:** 2015-02-28

**Authors:** Laura M Dale, Sophie Goudreau, Stephane Perron, Martina S Ragettli, Marianne Hatzopoulou, Audrey Smargiassi

**Affiliations:** McGill School of Environment, McGill University, Montréal, QC Canada; Direction de santé publique de Montréal, Montréal, Canada; Department of Civil Engineering, McGill University, Montréal, QC Canada; Département de santé environnementale et de santé au travail, Université de Montréal, Montréal, H3C 3J7 Canada; Institut National de Santé Publique du Québec, Montréal, Canada

**Keywords:** Noise, Socioeconomic status, Deprivation, Environmental equity, Burden

## Abstract

**Background:**

This study’s objective was to determine whether socioeconomically deprived populations are exposed to greater levels of environmental noise.

**Methods:**

Indicators of socioeconomic status were correlated with LAeq24h noise levels estimated with a land-use regression model at a small geographic scale.

**Results:**

We found that noise exposure was associated with all socioeconomic indicators, with the strongest correlations found for median household income, proportion of people who spend over 30% of their income on housing, proportion of people below the low income boundary and with a social deprivation index combining several socio-economic variables.

**Conclusion:**

Our results were inconsistent with a number of studies performed elsewhere, indicating that locally conducted studies are imperative to assessing whether this double burden of noise exposure and low socioeconomic status exists in other contexts. The primary implication of our study is that noise exposure represents an environmental injustice in Montreal, which is an issue that merits both investigation and concern.

**Electronic supplementary material:**

The online version of this article (doi:10.1186/s12889-015-1571-2) contains supplementary material, which is available to authorized users.

## Background

Chronic exposure to noise has been linked to various adverse effects such as annoyance, sleep disturbance, impaired cognitive performance, as well as to the onset of cardiovascular diseases [1]. Environmental noise is one of the most widespread sources of stress and discomfort in urban areas and few studies have assessed its association with social deprivation [2-5].

Deprivation is defined as “a state of observable and demonstrable disadvantage relative to the local community or the wider society or nation to which an individual, family or group belongs” [6]. Also, Townsend [6] describes two main forms of “relative” deprivation. The first, “relative” material deprivation refers to a deficiency of fundamental goods and conveniences such as a safe place to live, an adequate diet, and basic amenities. The second, “relative” social deprivation refers to a lack of adequate social relationships with members of one’s family, community, or workplace. While each form of “relative” deprivation may have its own public health implication, socioeconomic status is often used as an indicator of “relative” deprivation.

Living in disadvantaged communities can be deleterious for health as a result of any and all of at least five health-influencing characteristics described by Stokols [7]. As such, one’s environment may act as 1) a medium for disease transmission, 2) a stressor, 3) a source of safety or danger, 4) an enabler or hinderer of healthy behavior, and/or 5) a provider (or not) of health resources. Furthermore, poorer individuals are less empowered and may face fewer choices of where to live, often forcing them to reside in dwellings with inadequate conditions, and near a larger number of environmental stressors such as toxic waste dumps, industrial sites, and roads with high traffic density [8-10]. However, higher exposures to environmental stressors have also been noted in wealthier populations. For example, Cesaroni et al. [11] noted that individuals living in areas of high road traffic were of higher social position in Rome.

Few studies have assessed associations between socio-economic status and exposure to noise. The study conducted by Hoffmann et al. [12], in Germany, noted a negative correlation with noise pollution (from traffic noise) along the entire gradient of socioeconomic status based on four social indicators. Similarly in Hong Kong [2] and in the Twin Cities, Minnesota [5], socially disadvantaged groups were more exposed to traffic noise. In Paris (France), Havard et al. [3] reported that people living in advantaged neighborhoods were more exposed to road traffic noise than their less affluent counterparts. In Marseille (France), a non-linear relationship, with the highest exposure to road traffic noise at a middle level socio-economic status was noted [4]. Thus, studies on such trends are scarce and methodologically inconsistent, and have yet to be done in Montreal.

The objective of the present study was to determine whether there is a correlation between the socioeconomic status of populations in Montreal and exposure to environmental noise.

## Methods

### Study area

**Figure 1:**
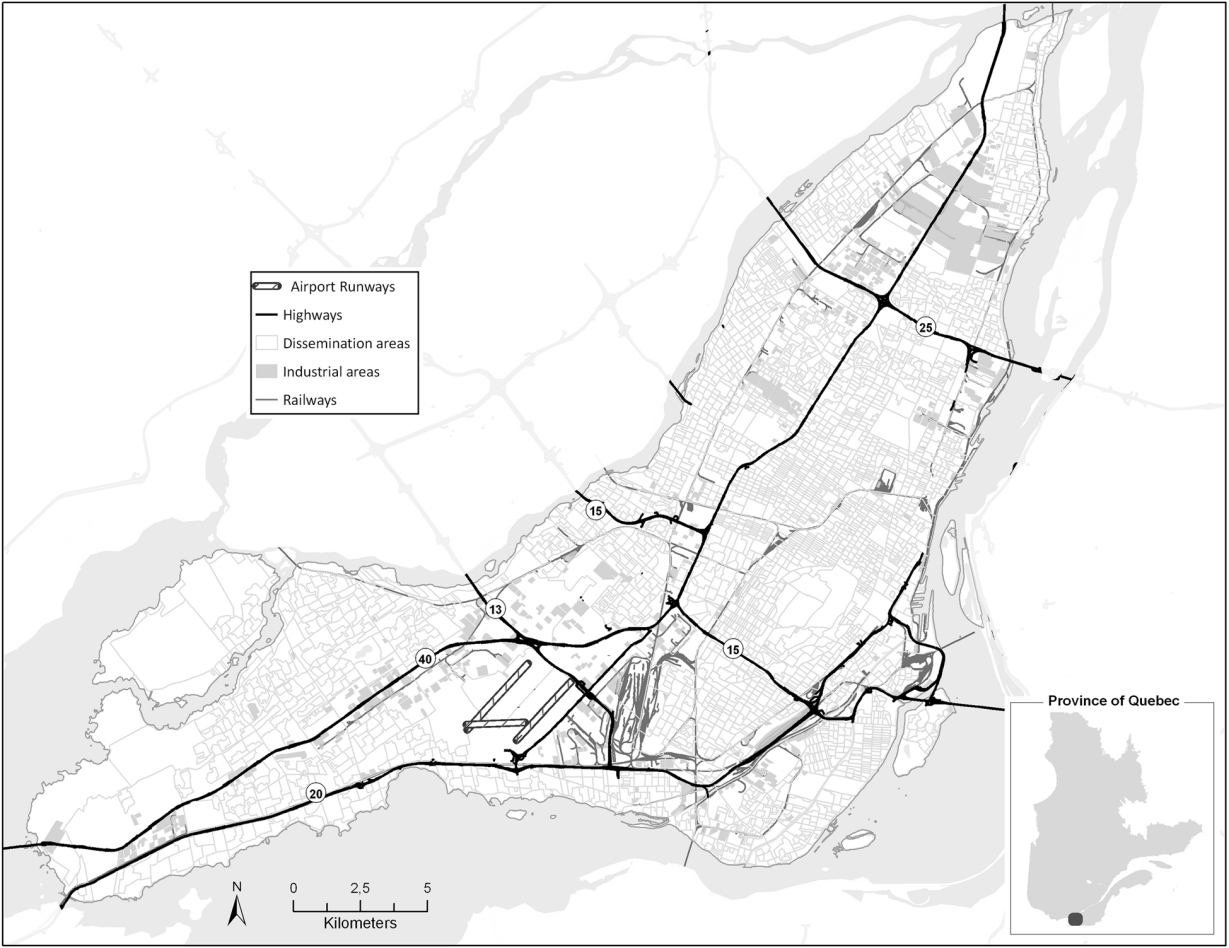
**Map of Montreal Island.**

Our study took place on the island of Montreal, where we examined neighborhood scale social and physical environmental characteristics. The island has an area of 500 km^2^ that contains 19 boroughs. Road traffic among the several expressways throughout the city is heavy, particularly along the number 13, 15, 20, 25 and 40 highways, which span the whole island. A number of railway tracks also reach an extensive portion of the island. There exists a large international airport in the Dorval region (to the West of the Island), and in certain areas, particularly in the eastern portion of the city, there are clusters of industrial activity. Whereas the most densely populated areas are located around the city center and between highways 13 and 25, the territory west of highway 13 is more suburban in character with low residential density; the area east of highway 25 is mainly of low and medium residential density (Figure 1).

Montreal differs from many other large North American cities in a number of ways. For example, visible minority or immigration statuses tend not to correlate to a great extent with low socioeconomic status [13,14]. Regardless, Montreal represents one of the most economically segregated cities in Canada, with the least equal income distribution for the year 2000, as determined by the median share of income received by the less well-off half of the population [15]. This inequality translates into real health disparities, for example, a variation in life expectancy among men in different parts of the city by six years [16]. A more detailed description of the physical and social geography of Montreal can be found in Crouse et al.’s study on air pollution and socioeconomic deprivation [17].

### Socioeconomic indicators

The socioeconomic characteristics of Montreal’s population were described using 2006 census data from Statistics Canada aggregated at the dissemination area level. Although more recent data is available (i.e. 2011), the validity of the last Canadian census data has been questioned given that the questions related to the socio-economic status are, since 2011, provided on a voluntary basis and thus not mandatory as in the past [18].

Dissemination areas are the smallest standard geographic areas for which all census data are distributed and they respect several delineation criteria. They 1) respect the boundaries of census subdivisions and census tracts, allowing them to remain fairly stable over time, 2) follow linear features such as roads, 3) are uniform in terms of population size, containing 400–700 persons (larger or smaller sizes may result in order to respect criterion 1), 4) are delineated based on block population counts from the previous census, and 5) are compact in shape as much as possible [19].

The dataset included 3147 dissemination areas with a population greater than zero. 28 dissemination areas were without a population, due to their location in industrial areas or in large parks such as the Mount Royal Park. The area of the dissemination areas ranged from 0.07 km^2^ to 17.8 km^2^ and the mean was 0.16 km^2^. The populations of the 3147 dissemination areas considered ranged from 113 to 4877 and the mean was 585.

Eight indicators of socioeconomic status were studied for each dissemination area :1) proportion of households with only one person, 2) unemployment rate, 3) proportion of people over the age of 25 without a diploma, 4) proportion of people below the low-income boundary, 5) median household income, and 6) proportion of people who spend over 30% of their income on housing. Furthermore, two indicators combining several socio-economic variables and developed by Pampalon and Raymond [20] were used, 7) the material deprivation index and 8) the social deprivation index. Each of these indicators has been used regularly in studies linking deprivation with health outcomes [17,20,21].

### Noise levels

A-weighted outdoor summer noise levels (LAeq24h) were computed for cells of 20 m × 20 m on the Island of Montreal, using a Land Use Regression (LUR) model [22]. This LUR model was developed based on LAeq24h from a two-week sampling period at 87 sites during the summer 2010, along with determinants of the built environment in Montreal (e.g. vegetation, land use, road network, etc.). These determinants focused on transportation and industrial noise sources, but did not include all neighborhood outdoor noise sources such as bars, parking, etc. We assigned, to each dissemination area, the average of all summer noise level estimates within that area.

### Statistical analyses

The strength and the direction of the relation between mean predicted dissemination area LAeq24h noise levels and each of eight indicators was assessed by computing Pearson correlation coefficients.

### Map

A map illustrating the double burden of noise exposure and deprivation was produced as follows. First, quintiles of noise levels for the dissemination areas and quintile of each the indicators of the socioeconomic status were calculated; the fifth quintile represented the worst noise or deprivation level. Then, the values from one to five, for the noise and the value from one to five for each socioeconomic indicator were added for each dissemination area. Thus dissemination areas with a value of ten represented both the worst noise and lowest socioeconomic status.

## Results

**Figure 2:**
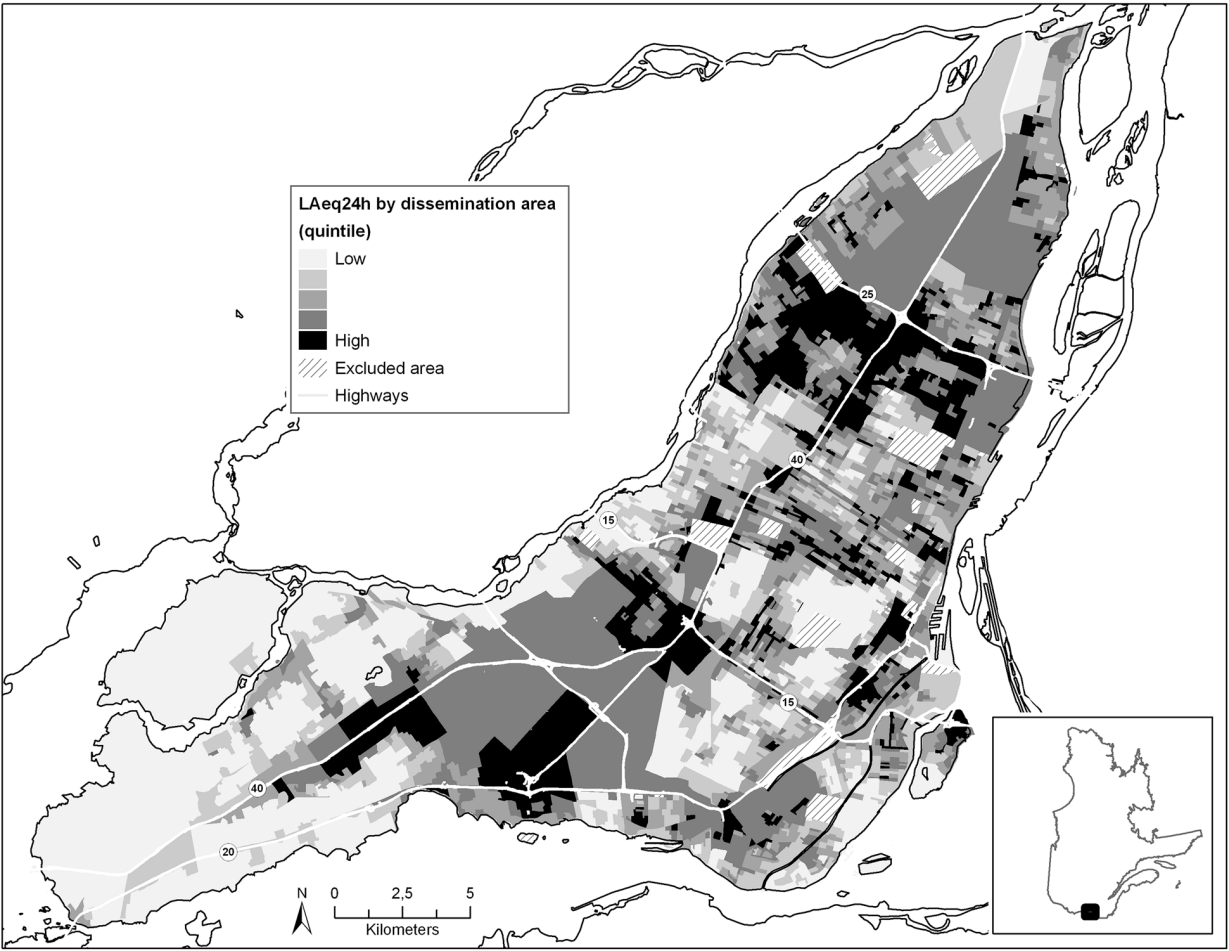
**LAeq24h noise levels by dissemination areas in Montreal.**

LAeq24h noise levels for the sampling period were in the range of 50.5-68.8 dBA with an arithmetic mean of 58.3 ± 3.2 dBA and median level of 58.3 dBA. The Island of Montreal is noisier mainly where highways and industrial areas are present in the north-east of the island, and in the west where highways and the international Montreal Airport are also found (Figure 2).

The Pearson correlation coefficients for LAeq24h levels with the indicators of the socioeconomic status were in the range of |0.232| to |0.426|, all in their expected direction so as to indicate a relationship between socioeconomic deprivation and elevated noise exposure (Table 1). Correlations were usually linear, as represented in the scatter plots (Additional file 1: Figure S1), except for median income, which approximated a negative exponential relation with noise levels.

**Table 1:** **Descriptive statistics of dissemination area variables (n = 3147) and their correlations with noise levels**

**Variables (by dissemination area)**	**Mean**	**Median**	**Standard deviation**	**1st percentile**	**99th percentile**	**Pearson correlation with mean LAeq24h**	
						**Coefficient**	**p value**
Proportion of households with 1 person	34.7	35.3	14.8	4.3	68.2	0.259	<0.0001
Unemployment rate	8.8	7.7	6.3	0	28.6	0.232	<0.0001
Proportion of people over age 25 without a diploma	20.8	19.0	12.4	0	50.0	0.311	<0.0001
Proportion of people below the low income boundary	21.8	20.0	14.7	0	61.8	0.360	<0.0001
Median household income*	47 735	40 420	26 263	17 006	147 114	−0.426	<0.0001
Proportion of people who spend over 30% of their income on housing	21.2	20.5	14.3	0	57.4	0.378	<0.0001
Material deprivation index	−0.003	−0.004	0.048	−0.116	0.112	0.380	<0.0001
Social deprivation index	0.014	0.020	0.043	−0.097	0.100	0.337	<0.0001

*Distributions of income, after tax and earnings distributions have been suppressed where the total number of units (persons, families or households) in the reference year estimated number is less than 250.

**Figure 3:**
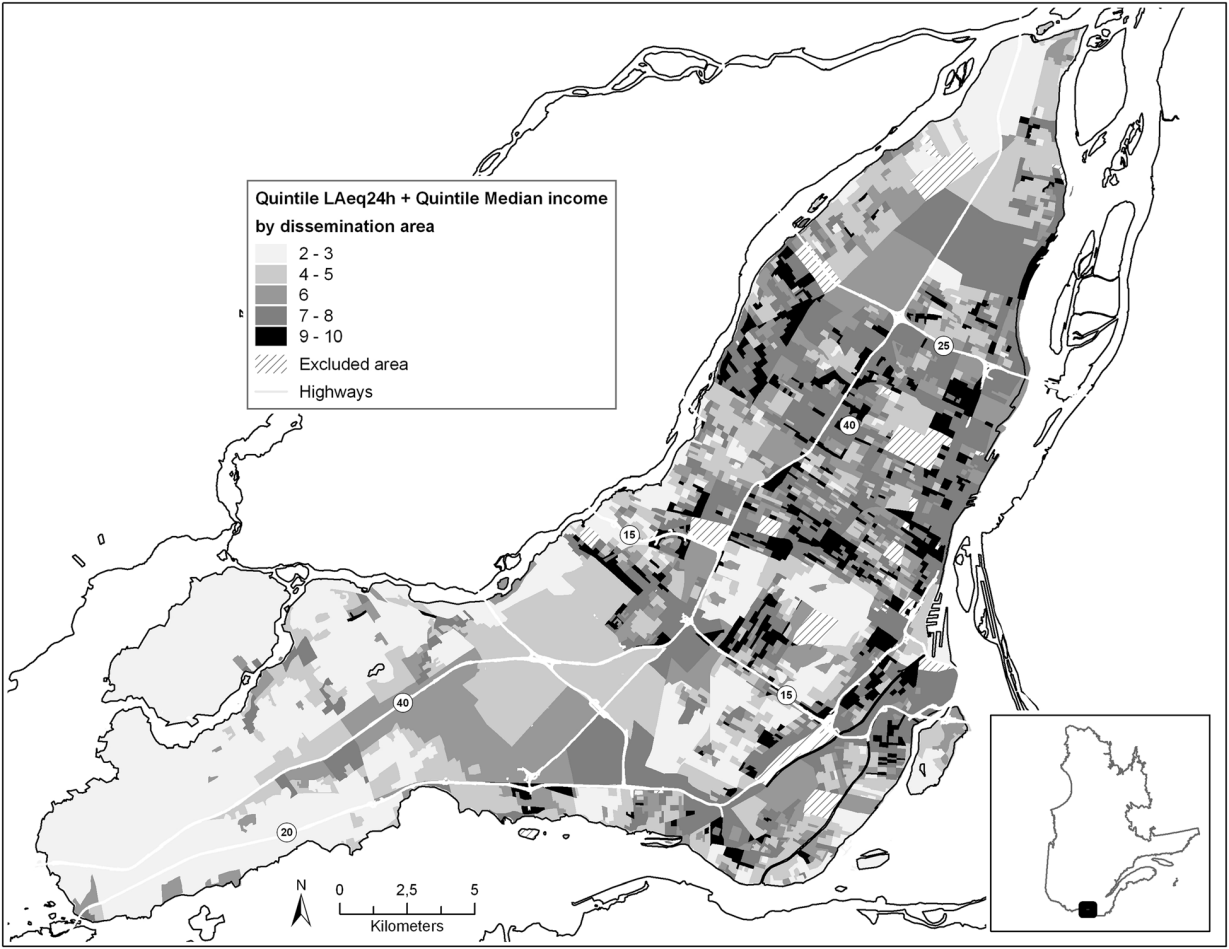
**Sum of quintile values of noise and median household income by dissemination areas in Montreal.**

Figure 3 presents a map that combines the sound levels and the median household income. The areas presenting a double burden of noise exposure and lowest household income are presented in black in the figure. 15.8% (n = 501) of dissemination areas presented a double burden (class 9 and 10). These dissemination areas were mainly found in the center of the Island of Montreal, although not really clustered in one location. Noise levels were low and median incomes were high in the dissemination areas in the west of the Island (Additional file 2: Figures S2). Similar results were obtained with other indicators of the socioeconomic status (maps not shown). For example, using the unemployment rate which is the socioeconomic indicator the least correlated with noise levels, we noted that 15.3% of dissemination areas presented a double burden (data not shown).

The information from the map is also summarized in Table 2. There were few dissemination areas affected by a single factor: 43 dissemination areas presented only the worst average income and 37 dissemination areas, the worst noise levels.

**Table 2:** **Number of dissemination areas by quintiles of median household income and average noise levels (LAeq24h), Montréal, 2006**

**Median household income by dissemination area (quintile)**	**Average LAeq24h by dissemination area (quintile)**					**Total**
	**1 (low)**	**2**	**3**	**4**	**5 (high)**	
**1 (high)**	283	126	121	68	37	635
**2**	100	129	159	140	106	634
**3**	56	135	143	162	138	634
**4**	38	97	179	169	150	633
**5 (low)**	43	74	143	163	188	611
**Total**	**520**	**561**	**745**	**702**	**619**	**3147**

## Discussion

In this study we found that environmental noise exposure appears to be higher in areas of greater socioeconomic disadvantage. Associations between noise and socioeconomic status indicators were moderate (<0.5) and usually linear.

Our results are consistent with those of a few studies that showed that increased noise levels are associated with decreased socioeconomic status [2-5,12-23]. However, the heterogeneity of results in other locations makes it clear that the present study is inconsistent with a number of others. For example, in Paris, France, Havard et al. [3] associated higher noise exposure from road traffic noise with higher socioeconomic status. A case study in the Rijnmond region of the Netherlands by Kruize and Bouwman [23] similarly associated higher income levels with higher noise exposure levels, except in the case of aircraft noise for which opposite trend was observed. In Marseilles, France, Bocquier et al. [4] found a non-linear relationship between social inequalities and exposure to road traffic noise, with the highest levels of exposure at a middle level of deprivation. Brainard et al. in Birmingham, UK [24] reported inconclusive trends between noise exposure from combined sources (road, rail and airport) and deprivation.

There are a number of considerations that should be taken into account when comparing our study with others. First, our study did not differentiate between different noise sources, or between nighttime and daytime noise. Second, we used different indicators for socioeconomic status than each of the studies described above (although overlap did occur).

Third, it should be noted that each of the studies mentioned above was conducted in Europe, where cities are frequently configured differently than in North America. Indeed the inner city in Western Europe is often wealthier than the suburbs whereas in Montreal, the proportion of people with a low socioeconomic statusis higher in the center of the Island, where higher noise levels can be found. Each of these considerations limits the comparability of our results.

Disregarding these methodological differences however, it would appear that the relationship between socioeconomic status and noise exposure is highly variable and dependent on local contexts. There is a need for further research in this field to reconcile and provide explanations for these regional discrepancies. By identifying explanatory factors, we may then begin to extrapolate local results to similar areas elsewhere in the world.

A study done in New York City on noise, air pollutants and traffic suggested that an imperfect correlation exists between noise and air pollution in the context of road traffic [25]. Comparing the results from the present study on noise and socioeconomic status with those from Crouse et al. [17] on air pollution and socioeconomic status, it is possible that such a discrepancy may also exist in Montreal. Crouse et al. [17] demonstrated a positive correlation between air pollution and socioeconomic status but with notable deviations from the overall trend in certain neighborhoods and for certain indicators. For example, the low education indicator was inconsistent with the idea of a double burden of socioeconomic disadvantage and air pollution exposure. In addition, two other indicators (proportion of lone-parent families and proportion of adults separated, divorced, or widowed) were non-linearly associated with air pollution exposure. Finally, the proportion of adults separated, divorced, or widowed had a positive association with air pollution until a specific point, at which the relationship became negative. Conversely, for each indicators used in our study, a double burden of low socioeconomic status and higher noise exposure was observed. Apparently, noise and air pollution are not uniformly linked in Montreal.

Our work is imperfect for a number of reasons. First, since it is impossible to measure noise everywhere, anytime and for the entire population, we used a land use regression model that predicts average sound levels over 24 hours (LAeq24h), with an error associated with the prediction of LAeq24h of 3.3 dBA.

Second, LAeq24h may not be the best indicator of exposure as sporadic noise events, such as those coming from aircrafts, buses or trucks, may be more disruptive than more constant streams of noise [26,27]. In addition, the 24 hr indicator does not allow us to differentiate between nighttime and daytime noise. Given the numerous potential effects of sleep disturbance on health, the possibility of certain areas having higher nighttime noise represents an important consideration. Similarly, our analysis considered only noise levels at people’s places of residence. Since not all people are home all day, they could have different overall exposure profiles than those suggested by our results.

Our results are also limited to outdoor noise levels; it is conceivable that a comparison of indoor noise would further emphasize this double burden. For example, low-income dwellings may be more likely to have features such as thinner windows and ineffective insulation, making them more susceptible to permeation by noise. Furthermore, the noise model by Goudreau et al. [22] used in this study is based on two-week measurements in the summer only, and does not consider potential seasonal differences due to weather conditions and noise sources. Nevertheless, in our previous work in Montreal, we noted a relatively good correlation between winter and summer noise levels (Pearson r = 0.74; Goudreau 2014, personal communication).

Furthermore, our assessment of the spatial distribution of the socioeconomic position is imperfect as we used aggregated socioeconomic indicators at the dissemination area level. Small-scale spatial variations of the socioeconomic status within a dissemination area may not be detected. It is indeed worth noting that concentrated pockets of individuals with low socio-economic status are generally small in Montreal, rarely covering an entire dissemination area [28].

Another consideration in our study design is the modifiable areal unit problem (MAUP), which arises due to the arbitrary nature by which the boundaries of areal units are chosen. If different boundaries were used instead (i.e. if the areal units were modified), then the results based on these delineations could be quite different [29]. Bowen [30] suggests that the appropriate level of spatial aggregation varies based on the objectives of the study, and that studies on exposure to environmental hazards call for smaller geographic units. The logic for this recommendation follows from spatial concerns regarding areal unit definitions. For example, it is conceivable that two populations on the boundaries of their respective areal units may be more similar to each other than to the populations within their own units. Also, given that dissemination areas are larger outside the city core, the error that can result from attributing the same noise levels to all individuals in a dissemination area may be greater outside the metropolitan center. The logical solution here would be to use a smaller spatial unit that better represents exposure [30]. We thus chose the smallest census unit available, dissemination areas.

A final consideration in our analysis is that the census data used in our study was from the year 2006 while our noise data was obtained in 2010. It is possible that the socioeconomic and geographic context has changed since then, although we do not have reason to believe so.

## Conclusion

The main implications of the present study are two-fold. First, it is apparent that studies linking environmental exposures to socioeconomic status require careful examination in local contexts. This finding arose in light of the many results on noise exposure and social conditions that have proven difficult to generalize to other areas. There is an apparent lack of environmental equity on the Island of Montreal. Our results indicate that deprived groups endure a double burden of low economic status and higher exposure to environmental noise. These findings thus highlight a societal externality imposed on an already disadvantaged group. Interventions to reduce noise levels in Montreal should be targeted to lower income neighborhoods.

## Additional files

Additional file 1: Figure S1. Scatterplots correlating LAeq24h with socioeconomic indicators. Additional file 2: Figure S2. Median household income by dissemination areas in Montreal.
