# Supplementary material for: Socioeconomic status and environmental noise exposure in Montreal, Canada
Source: BMC Public Health. 2015 Feb 28;15:205. doi: 10.1186/s12889-015-1571-2 (PMC4358710; doi:10.1186/s12889-015-1571-2)
Supplement: Additional file 1: Figure S1. — Scatterplots correlating LAeq24h with socioeconomic indicators. [file 12889_2015_1571_MOESM1_ESM.pdf]

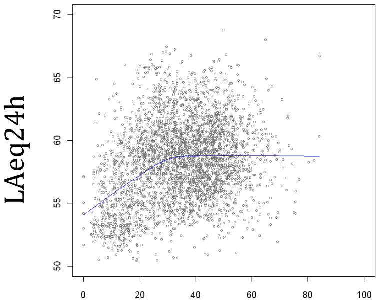

Proportion of households with 1 person

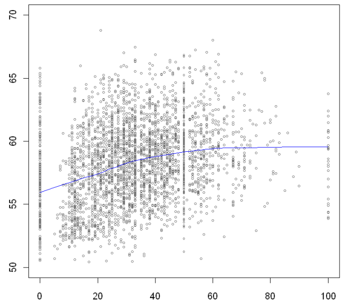

Unemployment rate

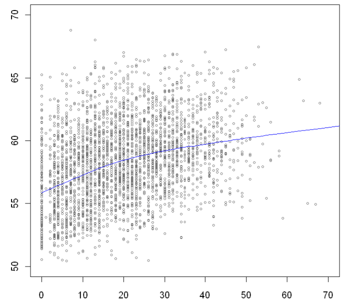

Proportion of people over age 25 without a diploma

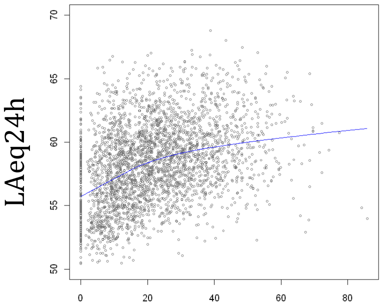

Proportion of people below the low income boundary

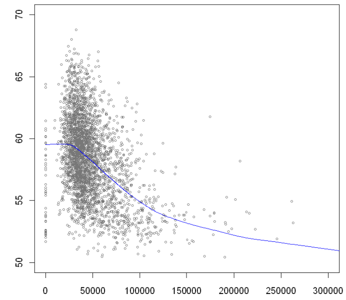

Median household income

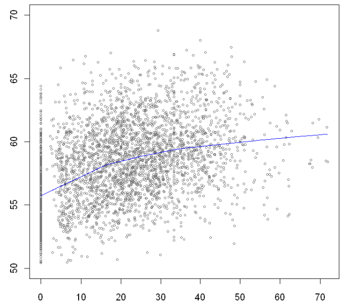

Proportion of people who spend over 30 % of their income on housing

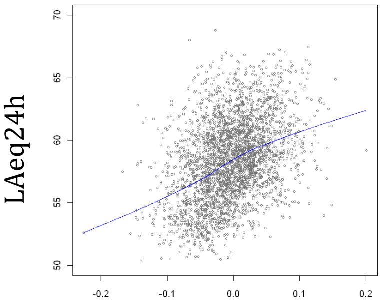

Material deprivation index

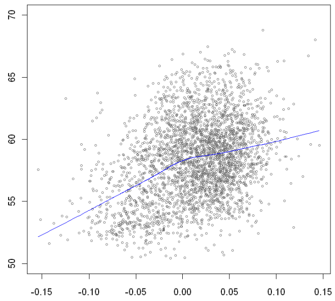

Social deprivation index
