# Supplementary figures and images for: Socioeconomic status and environmental noise exposure in Montreal, Canada
Source: BMC Public Health. 2015 Feb 28;15:205. doi: 10.1186/s12889-015-1571-2 (PMC4358710; doi:10.1186/s12889-015-1571-2)

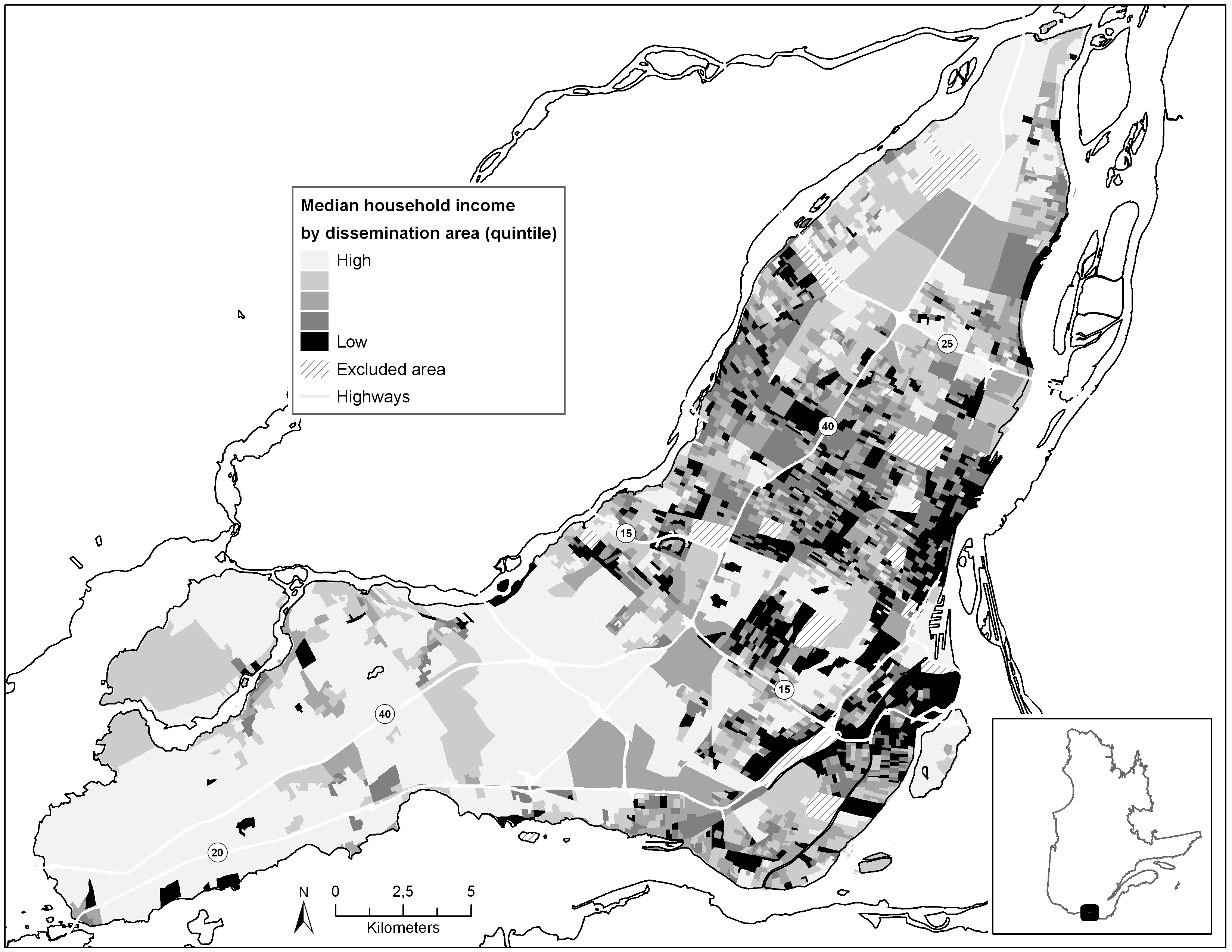

Supplement: Additional file 2: Figure S2. — Median household income by dissemination areas in Montreal. [file 12889_2015_1571_MOESM2_ESM.jpeg]
